# Beyond Gatekeeping: SIPAT as a Guide to Psychosocial Prehabilitation in a Single-Center Cohort of Lung Transplant Candidates

**DOI:** 10.3390/jcm15124487

**Published:** 2026-06-10

**Authors:** Aleksandra Stańska, Wojciech Karolak, Sławomir Żegleń, Jacek Wojarski

**Affiliations:** 1 Division of Quality of Life Research, Department of Psychology, Faculty of Health Sciences, Medical University of Gdańsk, Marii Skłodowskiej-Curie Street 3A, 80-210 Gdańsk, Poland; 2 Department of Cardiac & Vascular Surgery, Faculty of Medicine, Medical University of Gdańsk, 80-210 Gdańsk, Poland; 3 Division of Pulmonology, Faculty of Medicine, Medical University of Gdańsk, 80-210 Gdańsk, Poland

**Keywords:** lung transplantation, SIPAT, psychosocial assessment, prehabilitation, candidate selection, substance use

## Abstract

**Background:** Psychosocial assessment is central to lung transplant evaluation. Structured tools such as the Stanford Integrated Psychosocial Assessment for Transplantation (SIPAT) can be used either to support exclusionary decisions or to guide psychosocial prehabilitation by identifying modifiable targets for intervention. We examined how SIPAT functions in a program that explicitly prioritizes remediation of modifiable psychosocial risks. **Methods:** We conducted a retrospective observational cohort study of consecutive adult lung transplant candidates evaluated at a single center in Poland between December 2021 and November 2025. Psychosocial risk was assessed using SIPAT (locally translated), including total and domain scores, candidate categories, and binary indicators of clinically relevant alcohol, illicit substance, and nicotine-related risk. The primary endpoint was a pragmatic program outcome, defined as ever being listed (including transplanted) versus not being listed. Analyses focused on describing psychosocial risk profiles and their relationship to the program pathway rather than on building a predictive model of listing decisions. **Results:** In 491 candidates (mean age 57.2 years; 40.5% women), psychosocial burden was generally low (mean total SIPAT 12.4, SD 6.8), and most patients were rated as excellent or good candidates. SIPAT total, domain scores, and candidate categories were not meaningfully associated with ever being listed. Substance-related risk indicators were not independently associated with listing status after adjustment for age and sex. Cluster analyses based on SIPAT domain scores identified a higher-risk psychosocial profile, but cluster membership was not associated with listing status. ROC analyses showed that neither SIPAT total score nor domain scores discriminated listed from non-listed candidates, supporting the interpretation that SIPAT was not used as a binary gatekeeping tool in this program. **Conclusions:** In this prehabilitation-oriented program, SIPAT did not operate as a binary gatekeeping instrument for listing. Instead, it primarily served to identify modifiable psychosocial targets that trigger tailored support. These findings support using SIPAT as a structured roadmap for psychosocial prehabilitation rather than a stand-alone exclusion tool.

## 1. Background

Lung transplantation is a life-saving treatment option for selected patients with end-stage respiratory disease, but it remains constrained by donor organ scarcity and a high burden of post-transplant complications [1,2]. International guidelines, therefore, emphasize careful candidate selection that integrates medical, surgical, and psychosocial factors in order to maximize benefit while using organs responsibly [2]. Within this framework, psychosocial assessment has become a core component of transplant evaluation, particularly because potentially modifiable factors such as adherence, substance use, and social support can strongly influence outcomes [3,4,5].

Beyond these medical and organizational considerations, lung transplantation represents a profound psychological transition for patients with end-stage respiratory disease. Candidates often enter the transplant pathway after years of progressive dyspnea, functional decline, dependence on oxygen therapy, reduced autonomy, and uncertainty about survival. The evaluation and waiting-list period may therefore activate psychological mechanisms typical of life-threatening illness, including anticipatory anxiety, depressive symptoms, loss of control, illness-related uncertainty, altered identity, and dependence on caregivers and medical systems [6,7,8,9]. These reactions are not merely secondary emotional responses but may have clinically relevant implications for transplant readiness and long-term adaptation. They may influence motivation, illness understanding, treatment engagement, adherence, coping with waiting-list uncertainty, and the ability to participate in complex post-transplant self-management.

A large body of research in solid organ transplantation shows that nonadherence to medical recommendations and relapse to harmful substance use are common and have been linked to poorer clinical and functional outcomes, including higher morbidity and, in some studies, increased mortality [3,4,10,11,12]. However, psychosocial assessment practices have historically been heterogeneous, ranging from unstructured clinical impressions to locally developed checklists. This variability raises concerns about the consistency, transparency, and fairness of psychosocial decision making, including the risk that similar patients may be judged differently across centers or even across clinicians within the same centers [13,14,15]. In lung transplantation specifically, empirical studies have highlighted several psychosocial antecedents that may be relevant before and after listing, including psychological distress, depression, anxiety, lower resilience, limited social support, tobacco and alcohol use, and difficulties with coping during the waiting-list period [6,7,8,9,16,17,18]. These factors may affect transplant readiness through multiple pathways: reduced engagement with treatment recommendations, poorer communication with the transplant team, unstable caregiving arrangements, relapse to harmful health behaviors, and reduced capacity to tolerate uncertainty during prolonged evaluation or waiting.

The Stanford Integrated Psychosocial Assessment for Transplantation (SIPAT) was developed to address these limitations by providing a structured, theory-driven rating system for key psychosocial domains relevant to transplant candidacy and outcomes [19]. SIPAT covers four domains: readiness and illness management, social support system, psychological stability and psychopathology, and lifestyle and substance use. In contrast to purely categorical decisions, SIPAT yields both a continuous total score and risk categories that are intended to support multidisciplinary decision making, highlight modifiable risk factors, and guide pre-transplant interventions, rather than serve as a stand-alone pass or fail criterion [19].

From a psychological perspective, SIPAT is clinically relevant because it operationalizes several mechanisms that may shape adaptation to transplantation. The readiness and illness management domain captures illness insight, motivation, health literacy, and behavioral self-regulation. The social support domain reflects the availability and reliability of interpersonal resources needed for post-operative care, medication management, and crisis response. The psychological stability domain addresses current and past psychiatric symptoms, coping capacity, and emotional regulation. Finally, the lifestyle and substance use domain captures behavioral risks, including relapse vulnerability and the capacity to maintain abstinence in the context of chronic stress [6,19,20]. Thus, SIPAT should be understood not only as a risk score, but also as a structured map of potentially modifiable psychosocial mechanisms.

Among these mechanisms, alcohol use, nicotine use, and illicit substance use are of particular clinical relevance because they frequently represent explicit targets of transplant-related monitoring and intervention. Unlike broader psychosocial domains, these factors often require documented abstinence, addiction-focused treatment, smoking cessation programs, or ongoing behavioral monitoring before listing. Therefore, in addition to the composite lifestyle and substance use domain, these indicators were also examined separately in the present study.

Empirical work has increasingly evaluated the predictive utility of SIPAT across different organs [20,21,22,23]. In liver transplantation, higher SIPAT scores have been linked to poorer adherence and more complicated post-transplant courses in several studies [20,21], whereas other work has reported no clear association with key outcomes such as immunosuppressant adherence or rejection [23]. A recent single-center study in lung transplantation reported that higher baseline SIPAT scores were associated with a greater burden of medical and psychosocial complications during the first post-transplant year [24]. A narrative review of psychosocial assessment tools concluded that SIPAT is among the best studied instruments and may help identify patients at elevated risk of adverse psychosocial and behavioral outcomes, although effect sizes are often modest and findings are heterogeneous [13]. Together, these studies suggest that SIPAT captures clinically meaningful risk, but also highlight that its performance and clinical use may vary substantially across settings [13,19,20,21,22,23,24].

At the same time, international listing guidelines increasingly recommend that psychosocial risk factors should not automatically lead to exclusion from transplantation, especially when they are potentially modifiable through targeted intervention [2,25]. Instead, high-risk profiles are often viewed as indications for additional support, such as structured addiction treatment, intensified education about the transplant regimen, or efforts to stabilize the patient’s living situation [25,26,27,28]. Psychosocial prehabilitation can therefore be conceptualized as the psychological and behavioral counterpart of physical prehabilitation. Its goal is not simply to document risk, but to reduce modifiable vulnerabilities before transplantation. In practice, this may include psychoeducation, motivational interviewing, smoking cessation support, addiction treatment, psychiatric stabilization, adherence-focused counseling, caregiver involvement, and social work interventions aimed at strengthening the practical support system [6,26,27,28]. Although the evidence base for psychosocial prehabilitation in transplantation remains less developed than that for physical prehabilitation, existing lung transplant literature supports the clinical importance of addressing distress, coping, resilience, substance use, and social support before transplantation [6,7,8,9,16,17,18].

This raises an important conceptual tension. SIPAT can be used either as a gatekeeping tool that contributes to denying access to transplantation or as a support and flag tool that helps allocate psychosocial prehabilitation resources to vulnerable candidates while maintaining equitable access. Data on how SIPAT scores actually relate to listing decisions in real-world clinical practice are still limited. Existing studies have typically focused on post-transplant outcomes among patients who were already listed and transplanted, or have pooled different solid organ populations, making it difficult to draw conclusions specific to lung transplantation [13,19,20,21,22,23,24]. Moreover, most reports come from centers where high psychosocial risk frequently contributes to deferral or denial of listing, which may reinforce a more restrictive interpretation of the tool. There is a paucity of evidence from programs that systematically work with high-risk patients to remediate psychosocial vulnerabilities instead of using them primarily as exclusion criteria.

Our center has previously described the demographic and psychosocial characteristics of lung transplant candidates, including the distribution of SIPAT scores and domains in a single center cohort [29]. That analysis showed a wide spectrum of psychosocial risk, with a sizeable subgroup presenting with relevant substance use histories, psychiatric comorbidity, or fragile social support, but did not examine whether these factors translated into different listing outcomes. Building on that work, the present study focuses specifically on the relationship between SIPAT scores, substance use risk indicators derived from SIPAT, and subsequent listing status in a program that explicitly treats SIPAT as an entry point into psychosocial prehabilitation rather than a definitive barrier to transplantation.

In a lung transplant program that aims not to “disqualify for life” on psychosocial grounds but to identify and address modifiable risks, it is not obvious whether higher SIPAT scores should still predict a lower probability of being listed. One possibility is that structured psychosocial prehabilitation attenuates the impact of baseline psychosocial risk, so that candidates with initially elevated SIPAT scores may still progress to listing after targeted interventions. Alternatively, SIPAT may function more like a traditional gatekeeping instrument, with higher-risk profiles being less likely to reach the waiting list despite available support.

Therefore, the present study addressed three research questions. First, what psychosocial risk profiles are observed among adult lung transplant candidates assessed with SIPAT in a single-center prehabilitation-oriented program? Second, are SIPAT total scores, domain scores, global risk categories, and substance-use indicators associated with subsequent listing status? Third, do the observed patterns support the interpretation of SIPAT primarily as a gatekeeping instrument, or rather as a structured tool for identifying psychosocial prehabilitation needs and allocating support?

## 2. Methods

### 2.1. Setting and Participants

This retrospective observational cohort study included consecutive adult candidates for lung transplantation who were admitted for the first time to either the Lung Transplantation Unit or the Clinic of Pulmonology at the University Clinical Center in Gdańsk, Poland, between December 2021 and November 2025. During this initial hospital stay, all patients underwent a standard pre-transplant work-up and were discussed at the center’s multidisciplinary lung transplant board.

All consecutive adult patients admitted for an initial lung transplant evaluation during the study period were considered for inclusion. The clinical pathway was identical for all candidates: admission for transplant work-up, routine psychosocial consultation, SIPAT scoring by the clinical psychologist, preparation of a psychosocial summary, and multidisciplinary discussion by the transplant board. Data for the present study were extracted retrospectively from medical records and board documentation after completion of routine clinical evaluation.

In our program, psychosocial assessment is an obligatory component of the first inpatient transplant evaluation. All patients admitted for lung transplant work-up are routinely referred for consultation with a clinical psychologist. For the present analyses, we included all individuals who had a complete psychosocial evaluation and a fully scored SIPAT form. Repeated evaluations, readmissions, and incomplete SIPAT protocols were excluded. Basic sociodemographic variables (age, sex) and primary pulmonary diagnosis were retrieved from medical records.

Psychosocial eligibility was evaluated with SIPAT as part of routine clinical care. For this study, SIPAT ratings and clinical data were analyzed retrospectively in anonymized form only. No procedures beyond standard care were introduced, and no additional written informed consent was obtained. The project was reviewed according to institutional procedures for retrospective analysis of anonymized routine clinical data.

### 2.2. Psychosocial Assessment and Prehabilitation Approach

Psychosocial evaluations were carried out by clinical psychologists with experience in transplantation and chronic lung disease. Each assessment combined a semi-structured clinical interview, review of the medical chart, and, when clinically useful, discussion with the treating team or family.

The consultation focused on illness understanding, history of treatment adherence, health-related behaviors, psychiatric symptoms, coping strategies, social support, and substance use. Based on all available information, the psychologist completed the SIPAT rating and prepared a concise psychosocial report for presentation at the lung transplant board.

Importantly, in this program, SIPAT is embedded in a psychosocial prehabilitation framework rather than a purely exclusionary one. Candidates who present with elevated psychosocial risk in specific domains are routinely offered targeted interventions before a final listing decision, including structured smoking cessation support, addiction treatment, psychiatric care, and social work input aimed at stabilizing housing or caregiving arrangements [26,27,28]. In other words, high SIPAT scores trigger psychosocial prehabilitation efforts rather than an automatic decision not to list. No research-specific questionnaires or experimental procedures were added.

### 2.3. Stanford Integrated Psychosocial Assessment for Transplantation (SIPAT)

Psychosocial risk was assessed using the Stanford Integrated Psychosocial Assessment for Transplantation (SIPAT), developed by Maldonado and colleagues as a structured clinician-rated tool to capture psychosocial factors relevant for transplant candidacy and outcomes [19,20].

The instrument groups items into four domains:

**Readiness and illness management** (for example, illness insight, treatment adherence, lifestyle factors, motivation for transplantation);

**Social support system** (living arrangements, availability, reliability, and stability of caregivers);

**Psychological stability and psychopathology** (current and past psychiatric symptoms, coping style, personality factors);

**Lifestyle and substance use** (alcohol, nicotine, and other psychoactive substances).

Items are scored on ordered categorical scales, with higher values indicating higher psychosocial risk. For each patient, we calculated the following:

- The total SIPAT score (sum of all items);
- Four domain scores (sums of items in domains A–D);
- The original SIPAT global risk category (excellent, good, minimally acceptable, poor, or high-risk candidate) [19].

At the time of the study, no officially validated Polish version of SIPAT was available. In our center, we use a locally prepared Polish translation, developed by a team of psychologists and physicians fluent in English. The wording was minimally adapted to the lung transplant context and reviewed by bilingual clinicians. This version is used in everyday practice but has not yet undergone full cultural adaptation and validation.

### 2.4. Substance Use Indicators

From the **Lifestyle and effect of substance use** SIPAT domain, we derived three binary indicators reflecting clinically relevant substance use risk:

- Alcohol-related risk (yes or no);
- Illicit substances-related risk (yes or no);
- Nicotine-related risk (yes or no).

Patients were coded as having risk present if the SIPAT rating reflected current or past alcohol, drug, or nicotine use at a level considered clinically significant in the original scoring guidelines; for example, harmful use, dependence, or a high estimated risk of relapse [19,20]. All remaining patients were coded as having no risk in that domain. These indicators were used both in descriptive analyses and as predictors in regression models.

### 2.5. Transplant Listing Status

For each candidate, we obtained the final decision of the lung transplant board from the multidisciplinary meeting records. Clinical status was coded into four mutually exclusive categories:

1. Transplanted;
2. Currently on the active lung transplant waiting list;
3. Definitively not listed (psychosocial or medical disqualification);
4. Temporarily suspended or deferred (for example, ongoing work-up, need for additional treatment, weight reduction, or further stabilization).

In a subset of patients for whom the board had already reached a definite decision regarding eligibility, we also distinguished a binary variable “qualified for listing” versus “not qualified”, regardless of whether transplantation had already been performed.

For the main analyses, we created a pragmatic binary outcome variable reflecting whether a patient had ever been listed for lung transplantation. This pragmatic outcome reflects progression within the evaluation pathway in a real-world program and should not be interpreted as a pure psychosocial decision endpoint, as listing decisions integrate dynamic medical, logistical, and multidisciplinary factors. Patients who were transplanted or currently on the waiting list were classified as listed, while those disqualified, temporarily suspended, or still undergoing extended evaluation without being placed on the list were classified as non-listed. This variable was used as the primary dependent variable for between-group comparisons and logistic regression.

### 2.6. Statistical Analysis

All analyses were conducted using IBM SPSS Statistics, version 23 (IBM Corp., Armonk, NY, USA), and R version 4.5.3 (R Foundation for Statistical Computing, Vienna, Austria). ROC analyses with DeLong confidence intervals, logistic regression diagnostics, and k-means cluster visualizations were performed in R. Two-sided *p* values below 0.05 were considered statistically significant. Given the exploratory and hypothesis-generating nature of the study, no formal correction for multiple testing was applied. Because this was a retrospective observational cohort including all consecutive eligible candidates with complete SIPAT data during the study period, no a priori power calculation was performed. The sample size was determined pragmatically by the number of available complete evaluations. The analyses should therefore be interpreted as exploratory and descriptive of real-world clinical practice rather than as a confirmatory trial designed to test a pre-specified effect size.

Continuous variables were inspected for outliers and distributional properties and are reported as mean and standard deviation (*M* ± *SD*) or, where appropriate, median and interquartile range. Categorical variables are presented as counts and percentages. Normality was assessed using the Shapiro–Wilk and Kolmogorov–Smirnov tests together with visual inspection of histograms. Because SIPAT total and domain scores deviated from a normal distribution, non-parametric tests were used for group comparisons involving these variables.

To describe the sample, we summarized age, sex, and primary pulmonary diagnoses, as well as SIPAT total scores, domain scores, global candidate categories, and the prevalence of clinically relevant alcohol-, drug-, and nicotine-related risk indicators derived from SIPAT domain D.

The primary outcome was transplant listing status at the time of data extraction, dichotomized as listed (ever on the active waiting list or already transplanted) versus non-listed. Continuous variables, including age, SIPAT total score, and SIPAT domain scores, were initially compared between listed and non-listed candidates using Mann–Whitney U tests, because SIPAT variables were not normally distributed. In exploratory analyses, ordinal SIPAT items reflecting alcohol use, illicit substance use, and nicotine dependence were also compared using Mann–Whitney U tests. These analyses were intended as descriptive, unadjusted comparisons only. The primary inferential analyses were based on multivariable logistic regression models adjusted for age and sex. Therefore, conclusions regarding associations with listing status were based primarily on adjusted regression analyses. Categorical variables, including SIPAT candidate categories, were examined using chi-square (χ^2^) tests and, where relevant, additionally evaluated using adjusted logistic regression models.

To examine categorical aspects of psychosocial risk, we analyzed the distribution of SIPAT global risk categories (excellent, good, minimally acceptable, poor, and high-risk candidates). For the main analyses, we contrasted candidates rated as excellent or good (categories 1–2) with those rated as minimally acceptable, poor, or high-risk (categories 3–5), and tested associations with listing status using χ^2^ tests.

To explore whether more complex psychosocial profiles could be identified while avoiding overlap between input variables, k-means cluster analysis was performed using standardized SIPAT domain scores. The SIPAT total score and individual substance-use items were not included as clustering variables because they overlap conceptually and statistically with the domain scores. A two-cluster solution was specified a priori for interpretability and to distinguish lower- versus higher-risk psychosocial profiles. Cluster profiles were examined and visually represented using mean domain scores. Associations between cluster membership and listing status were examined using χ^2^ tests.

Receiver operating characteristic (ROC) analyses were conducted to evaluate the discriminatory ability of SIPAT scores for transplant listing status. We calculated the area under the ROC curve (AUC) for SIPAT total score and, in exploratory analyses, for each SIPAT domain separately. Ninety-five percent confidence intervals for AUC values were calculated using DeLong’s method. These analyses were intended to examine whether SIPAT scores functioned as discriminators of listing decisions rather than as screeners for SIPAT-derived substance-use indicators.

To investigate predictors of being listed, we estimated binary logistic regression models with listing status (listed vs. non-listed) as the dependent variable. In response to the exploratory nature of the study and to improve transparency, we focused on a limited number of clinically interpretable models. Model 1 included age, sex, and SIPAT total score. Model 2 included age, sex, and the four SIPAT domain scores. Model 3 included age, sex, and binary indicators of alcohol-, nicotine-, and illicit substance-related risk. An additional model included age, sex, primary diagnosis category, and SIPAT total score to partially account for clinical heterogeneity. Odds ratios (OR) with 95% confidence intervals were reported for all predictors, alongside model fit indices and overall classification accuracy.

Secondary analyses focused on the subset of patients with a definitive qualitative decision about transplant eligibility. In this subgroup we created a binary variable reflecting “qualified” versus “not qualified” status (ever accepted or listed versus disqualified or definitively not listed). We then compared SIPAT domain and total scores between qualified and not qualified candidates using Mann–Whitney *U* tests and examined the distribution of SIPAT candidate categories across qualification status using χ^2^ tests.

## 3. Results

### 3.1. Sample Characteristics

A total of 491 lung transplant candidates were included in the analyses. The mean age was 57.20 years (*SD* = 10.73, range 19–78). Women constituted 40.5% (*n* = 199) and men 59.5% (*n* = 292). At the time of data extraction, 151 patients (30.8%) were listed (either actively on the waiting list or already transplanted), whereas 340 (69.2%) were not listed.

Detailed sociodemographic and clinical characteristics, including underlying diagnoses, are presented in Table 1.

### 3.2. SIPAT Scores and Listing Status (Listed vs. Non-Listed)

Psychosocial risk was assessed using SIPAT. The following components were analyzed: readiness and illness management, social support system, psychological stability and psychopathology, lifestyle and substance use, and the total SIPAT score. In the full cohort, the mean total SIPAT score was 12.38 (*SD* = 6.84, range 0–45). Most candidates were classified as excellent or good according to the original SIPAT categories.

SIPAT total and domain scores, global candidate categories, selected psychosocial risk indicators, and their distribution by listing status are presented in Table 2.

All SIPAT indices showed significant deviations from normality (Shapiro–Wilk, *p* < 0.001); therefore, non-parametric tests were applied. Comparisons between listed and non-listed candidates showed no significant differences for SIPAT total score, SIPAT domain scores, or SIPAT global categories (Table 2). Mean total SIPAT scores were nearly identical in both groups, indicating that overall psychosocial burden as measured by SIPAT was not associated with listing status in this cohort. Elevated alcohol-related risk was present in 4.5% of candidates, elevated nicotine-related risk in 11.4%, and elevated illicit-substance-related risk in 2.2% (Table 2).

For the main categorical analyses, SIPAT categories were collapsed into lower risk (excellent or good candidates, categories 1–2) versus higher risk (minimally acceptable, poor candidates, and high-risk candidates, categories 3–5). This dichotomized classification was then cross-tabulated with listing status (listed vs. non-listed). The association was non-significant (*Pearson chi-square*(1) = 0.10, *p* = 0.753; odds ratio for higher vs. lower risk ≈ 0.90, 95% CI [0.48, 1.70]), indicating that being categorized as a higher risk SIPAT candidate did not reduce the odds of being listed. This finding was consistent with the program’s prehabilitation-oriented approach, in which an elevated SIPAT category triggered additional psychosocial work rather than automatic exclusion. In the full cohort, only four patients were rated in the “poor candidate” category, and none of them were listed for transplantation. Given this very small number, these cases were not analyzed separately and are reported descriptively only. 

In an adjusted logistic regression model including age, sex, and dichotomized SIPAT risk category, the higher-risk SIPAT category was not associated with listing status (OR = 0.805, 95% CI 0.405–1.524, *p* = 0.520). Age remained the only significant predictor in this model (OR = 0.968, 95% CI 0.951–0.986, *p* < 0.001), whereas sex was not significant.

### 3.3. Cluster Analysis of Psychosocial Risk Profiles

**Figure 1:**
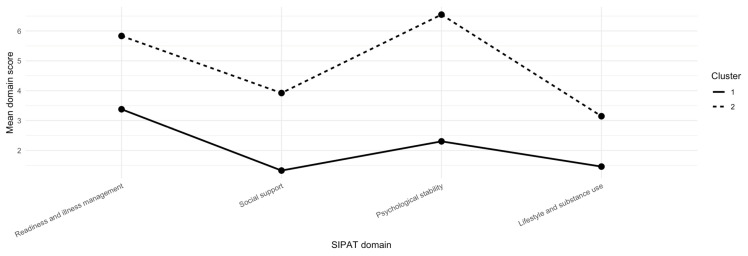
Psychosocial risk profiles identified using k-means clustering based on standardized SIPAT domain scores. Cluster 1 represents the lower-risk psychosocial profile and Cluster 2 represents the higher-risk profile.

To avoid overlap between input variables, k-means cluster analysis was performed using the four standardized SIPAT domain scores. This solution identified two clinically interpretable psychosocial profiles. Cluster 1 represented a lower-risk profile, with lower mean scores across all SIPAT domains: readiness and illness management (*M* = 3.38), social support (*M* = 1.32), psychological stability/psychopathology (*M* = 2.30), and lifestyle/substance use (*M* = 1.46). Cluster 2 represented a higher-risk profile, with elevated scores across all domains: readiness and illness management (*M* = 5.83), social support (*M* = 3.92), psychological stability/psychopathology (*M* = 6.55), and lifestyle/substance use (*M* = 3.15). The cluster profiles are presented in Figure 1.

Despite clear differences in psychosocial profiles, cluster membership was not associated with listing status. Among candidates in Cluster 1, 95 of 316 patients were listed, compared with 56 of 175 patients in Cluster 2. The association between cluster membership and listing status was non-significant (χ^2^ = 0.12, *p* = 0.731). This suggests that even candidates with a higher-risk psychosocial profile were not systematically excluded from the transplant pathway.

### 3.4. ROC Analyses: SIPAT Scores and Listing Status

**Figure 2:**
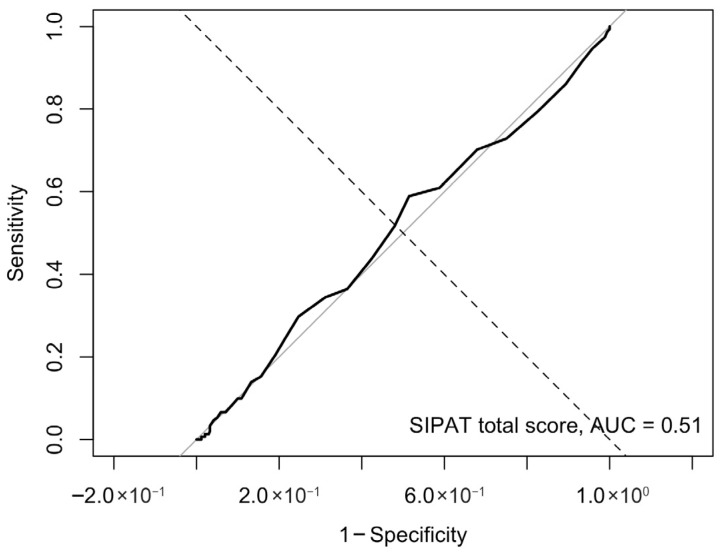
Receiver operating characteristic (ROC) curve for SIPAT total score discriminating between listed and non-listed candidates. The solid black line represents the observed ROC curve, the dashed diagonal line represents the line of no discrimination (AUC = 0.5), and the gray line represents an additional fitted trend line included for visualization. AUC = 0.51, 95% CI 0.45–0.56.

Receiver operating characteristic analyses demonstrated poor discriminatory ability of both the SIPAT total score and all four SIPAT domains for listing status (Table 3). As illustrated in Figure 2, the ROC curve for the SIPAT total score showed minimal discriminative ability, with an AUC of 0.51. All AUC values were close to 0.50, indicating performance no better than chance. These findings further support the interpretation that SIPAT scores did not function as a binary screening threshold for listing decisions in this cohort.

### 3.5. Logistic Regression Analyses of Factors Associated with Listing Status

Multivariable logistic regression models are presented in Table 4. In Model 1, adjusted for age and sex, SIPAT total score was not associated with listing status (OR = 0.995, 95% CI 0.967–1.024, *p* = 0.742). Age was the only significant predictor, with each additional year associated with lower odds of being listed (OR = 0.968, 95% CI 0.951–0.986, *p* < 0.001). Sex was not associated with listing status.

In Model 2, which included age, sex, and the four SIPAT domain scores, none of the SIPAT domains independently predicted listing status. Age remained the only significant predictor (OR = 0.968, 95% CI 0.951–0.986, *p* < 0.001).

In Model 3, binary indicators of alcohol-, nicotine-, and illicit substance-related risk were not significantly associated with listing status after adjustment for age and sex. Age again remained significant (OR = 0.966, 95% CI 0.948–0.983, *p* < 0.001).

In a sensitivity analysis additionally including primary diagnosis category, SIPAT total score remained non-significant. Across models, explained variance was low and classification accuracy ranged from 68.7% to 69.9%, largely reflecting the base rate of non-listing rather than meaningful discriminatory performance. This further supports the interpretation that psychosocial scores did not substantially improve discrimination of listing status.

Classification accuracy was calculated using a probability threshold of 0.50. Pseudo-R^2^ values indicate low explained variance across models.

### 3.6. Secondary Analysis: Qualified vs. Not Qualified Among Patients with Definitive Status

In the subgroup of 217 patients with a fully determined qualification outcome (171 qualified, 46 not qualified), Mann–Whitney tests were again used to compare SIPAT domain and total scores. Results mirrored the main analyses:

- No significant differences between qualified and not qualified patients in readiness and illness management, social support system, psychological stability and psychopathology, lifestyle and substance use, or SIPAT total (all *p* > 0.24);
- Substance-related scores (including nicotine, alcohol, and illicit substances) also did not significantly distinguish qualified versus non-qualified candidates;
- Age remained descriptively lower in qualified patients, consistent with the main logistic models.

The detailed cross-tabulation of SIPAT categories in this subgroup showed that minimally acceptable candidates were not at a higher risk of non-qualification. On the contrary, 83.3% of candidates in this higher risk category were accepted after the complete evaluation process, while no patients in the poor category were present in this subgroup. The association between SIPAT candidate category (excellent or good vs. minimally acceptable) and qualification status was non-significant in chi-square tests. This pattern is congruent with the center’s philosophy that minimally acceptable does not mean “too risky to touch” but “acceptable after psychosocial prehabilitation”.

## 4. Discussion

In this large single-center cohort of 491 lung transplant candidates, psychosocial risk as captured by the Stanford Integrated Psychosocial Assessment for Transplantation (SIPAT) was generally low and showed no meaningful association with listing decisions. Importantly, these findings should be interpreted within the context of a real-world, prehabilitation-oriented clinical program evaluation rather than as a formal predictive validation study designed to establish universal SIPAT cut-offs for transplant listing. Mean total SIPAT scores were almost identical in candidates who were ultimately listed (including transplanted and actively waitlisted patients) and those who remained non-listed (approximately 12 points in both groups), with no significant differences in any of the four SIPAT domains (readiness and illness management, social support, psychological stability/psychopathology, and lifestyle/substance use). Non-parametric comparisons and logistic regression models consistently indicated that age, rather than psychosocial risk, was the main predictor of being listed, with younger patients more likely to progress to listing (odds ratios per year around 0.96–0.97, *p* < 0.001).

The finding that age was the only consistent predictor of listing status should be interpreted in the context of existing lung transplant practice rather than as a new stand-alone selection rule. Age is already considered during multidisciplinary assessment in accordance with transplant guidelines, together with frailty, comorbidity burden, functional status, and expected post-transplant benefit. Therefore, this result does not imply that chronological age should be used as an isolated exclusion criterion. Rather, it confirms that listing decisions in this cohort were more strongly driven by established medical and eligibility considerations than by baseline psychosocial risk measured with SIPAT.

These findings should be interpreted in the context of prior lung transplant literature showing that psychosocial vulnerability is common but not uniformly predictive across outcomes. Previous studies have described psychological distress, depression, anxiety, substance use, coping difficulties, social support, and resilience as clinically relevant factors in lung transplant candidates and recipients [6,7,8,9,16,17,18]. For example, patients awaiting lung transplantation have been reported to experience elevated anxiety and depressive symptoms, and social support has been linked to psychological distress in candidates evaluated for transplantation [7,8]. Other studies suggest that resilience and affective functioning may be associated with transplant-related outcomes, including death or delisting before transplant [16,17]. Against this background, our results do not suggest that psychosocial functioning is irrelevant. Rather, they suggest that in a program designed to intervene on modifiable psychosocial risks, baseline SIPAT scores may not translate directly into lower listing probability.

ROC analyses further confirmed the absence of meaningful discrimination of listing status by SIPAT scores. The SIPAT total score and all four SIPAT domains showed AUC values close to 0.50. This pattern indicates that SIPAT captured psychosocial burden, as shown by the cluster profiles, but did not operate as a screening test separating candidates who were listed from those who were not.

Psychologically, the distinction between the lower-risk and higher-risk clusters should not be interpreted as a simple contrast between “safe” and “unsafe” candidates. Rather, it reflects different levels of pre-transplant support needs. Lower-risk candidates generally appear to have sufficient illness understanding, behavioral stability, emotional regulation, and interpersonal resources to proceed through the pathway with standard monitoring. Higher-risk candidates, by contrast, may require more intensive scaffolding before listing: repeated psychoeducation, closer adherence monitoring, psychiatric or addiction treatment, caregiver mobilization, or social work input. In this sense, the high-risk profile is best understood as a marker of psychosocial intervention needs and intervention intensity, not as a fixed psychosocial identity or an automatic contraindication.

Nicotine-related risk deserves specific clinical attention in lung transplantation because a substantial proportion of candidates have COPD or emphysema, conditions frequently linked to long-term tobacco exposure. In this cohort, nicotine-related risk was not independently associated with lower odds of listing after adjustment for age and sex. This is clinically important because it suggests that smoking history or relapse vulnerability did not function as a simple exclusionary marker. Instead, candidates with nicotine-related risk were typically directed toward cessation monitoring and support, and listing decisions were made in the context of demonstrated behavioral change and multidisciplinary judgment.

Overall, the findings are consistent with a model in which SIPAT functions as a descriptive, intervention-triggering tool rather than a binary gatekeeper. Psychosocial vulnerabilities are identified and addressed through what is essentially psychosocial prehabilitation, including targeted education, addiction treatment, social work, and psychological support, instead of being treated as permanent reasons for denial [26,27,28]. As a result, cross-sectional SIPAT scores do not “predict” being listed, because high-risk candidates remain actively engaged in the transplant pathway through structured psychosocial intervention and longitudinal support.

This interpretation is also consistent with a mechanism-based understanding of psychosocial risk. Many SIPAT components correspond to processes that are modifiable over time: knowledge can improve through education, adherence can be supported through routines and reminders, caregiver availability can be strengthened through structured planning, psychiatric symptoms can be treated, and substance-use risk can be reduced through monitoring and addiction-focused care. If these mechanisms are actively targeted, baseline psychosocial risk should not necessarily remain a stable predictor of listing. In a prehabilitation-oriented program, the expected clinical effect is precisely to weaken the direct association between initial risk and final access to transplantation.

### 4.1. Positioning Our Findings Within the SIPAT Literature

Most existing SIPAT studies have addressed a different question from ours. They typically examine whether higher pre-transplant SIPAT scores predict post-transplant outcomes such as immunosuppression non-adherence, rejection, hospital utilization, or mortality, rather than whether SIPAT predicts listing itself [13,19,20,21,22,23,24]. Maldonado and colleagues, who developed SIPAT, originally demonstrated that higher scores were associated with poorer adherence and worse psychosocial outcomes after transplantation, although associations with graft failure and mortality were more modest and variable [19,20]. Subsequent work in liver transplantation showed that higher SIPAT scores were related to greater risk of immunosuppression non-adherence and more complicated post-transplant courses [21], with partially similar patterns reported in kidney and kidney/pancreas recipients [22], although other studies have found no clear associations with key outcomes such as adherence or rejection [23], resulting in moderate effect sizes and heterogeneous findings overall.

In lung transplantation specifically, characterization studies have reported that SIPAT scores are often clustered in the “good candidate” range, with a smaller subgroup of clearly higher-risk patients, very similar to the two clusters we observed [29,30]. Hinton-Froese et al. found that higher SIPAT scores in lung recipients were associated with poorer 1-year psychosocial outcomes and aspects of medical complexity, but not with strong mortality signals [24]. A recent narrative review concluded that psychosocial tools such as SIPAT can modestly stratify risk but should not be interpreted as a standalone gatekeeping instrument [13].

This clustering in the “excellent” and “good” candidate range is clinically plausible for at least three reasons. First, patients who reach formal transplant evaluation have often already passed several layers of medical and behavioral preselection. Second, severe uncontrolled psychiatric illness, active substance dependence, or profound social instability may be addressed before referral or may prevent completion of the formal work-up. Third, lung transplant programs often monitor patients longitudinally, meaning that the SIPAT score captured during evaluation may already reflect some degree of stabilization, education, or behavioral change. Therefore, a low-to-moderate SIPAT distribution should not be taken to mean that psychosocial risk is absent, but rather that the formal candidate population is already partly selected and clinically managed.

Our findings extend this literature in several ways. First, we show in a large, unselected cohort of lung transplant candidates that SIPAT category, total score, and domain scores do not meaningfully differentiate candidates who are ultimately listed from those who remain non-listed. Chi-square tests for SIPAT categories and listing status were non-significant, and logistic regression models that included SIPAT total, domains, substance-use indicators, age, and sex improved model fit only minimally (Nagelkerke R^2^ ranged from 0.037 to 0.058). Classification accuracy was almost entirely driven by the base rate of non-listing and did not meaningfully improve once psychosocial variables were added.

The cluster analysis confirms that elevated psychosocial risk was concentrated in a clearly distinguishable higher-risk subgroup. However, ROC analyses showed that neither the SIPAT total score nor any SIPAT domain meaningfully discriminated listing status. Thus, SIPAT was able to characterize psychosocial burden, but this burden did not translate into a simple listing/non-listing gradient.

This is a crucial conceptual shift compared with settings in which SIPAT is used explicitly as a gatekeeping cut-off, for example, excluding candidates above thresholds such as 21 points [21]. In such systems, one would expect a clearer gradient between SIPAT scores and listing decisions [13,21,25,26,27]. In our data, the absence of such a gradient is not a failure of the instrument but rather a reflection of a different ethical and clinical philosophy: psychosocial risk is treated as a target for psychosocial prehabilitation rather than as a static contraindication.

### 4.2. SIPAT as a Roadmap for Psychosocial Prehabilitation Rather than a Binary Filter

The core implication of these findings is that SIPAT should not be reduced to a single cut-off that mechanically decides who gets listed. In a program where psychosocial services are explicitly integrated into the transplant pathway, SIPAT functions more as a roadmap for psychosocial prehabilitation [26,27,28]:

- The readiness and illness management domain identifies patients who need additional education, adherence support, or cognitive scaffolding;
- The social support domain highlights those who require structured involvement of caregivers, social work, or case management to ensure safe discharge and follow-up;
- The psychological stability domain marks individuals who need targeted psychiatric or psychotherapeutic input before and after transplantation;
- The lifestyle/substance-use domain signals those who should be prioritized for smoking cessation, alcohol treatment, or addiction services.

Several clinical examples illustrate why a single cut-off may be misleading. A candidate with a history of nicotine dependence may receive a higher SIPAT score, but if they demonstrate sustained abstinence, good illness understanding, strong caregiver support, and reliable follow-up, the clinically appropriate response may be continued cessation monitoring rather than exclusion. Conversely, a candidate with a relatively low total SIPAT score but poor illness insight or inconsistent adherence may require targeted education and close monitoring despite appearing “low risk” numerically. Similarly, a patient with anxiety or depressive symptoms may be psychosocially acceptable if symptoms are recognized, treated, and do not impair cooperation with the transplant regimen. These examples show why SIPAT is most useful when interpreted domain by domain and embedded in multidisciplinary clinical judgment.

### 4.3. Why Is SIPAT Not a Stronger Predictor of Listing in This Cohort?

Several features of our setting help explain why SIPAT is only weakly linked to listing probability:

1. **Restriction of range.** Average SIPAT scores were low, and most candidates fell into the “excellent” or “good” categories. As in other lung cohorts, this compression of scores inevitably reduces correlations with downstream decisions [24,29,30].
2. **Dynamic clinical decision making.** SIPAT is administered relatively early, whereas listing is a dynamic decision that incorporates changes in clinical status, psychosocial functioning, and response to interventions over time. A static baseline score can only imperfectly capture these moving targets.
3. **Deliberate decoupling of risk and exclusion.** The transplant team explicitly treats psychosocial problems as modifiable. Structural factors such as housing instability are addressed via social work and family support; psychiatric and addiction issues are treated and followed rather than treated as absolute contraindications [13,14,15,25,26,27,28]. By design, this weakens any cross-sectional association between initial SIPAT scores and ultimate listing.
4. **Medical factors dominate the final decision.** As in other studies, age and medical status remain the primary determinants of transplant suitability. In our analyses, age was by far the strongest predictor of being listed, which is consistent with current lung transplant guidelines [1,2].

### 4.4. Strengths and Limitations

Key strengths of this study include the large sample size, the specific focus on lung transplant candidates (a population still underrepresented in the SIPAT literature), and the use of SIPAT total scores, domain scores, dichotomized categories, binary risk indicators, ROC curves, and cluster analysis to characterize psychosocial risk. The fact that SIPAT was administered systematically to virtually all candidates enhances ecological validity; we are not dealing with a highly selected sub-cohort. The analyses directly reflect how SIPAT operates in a real-world, multidisciplinary program that explicitly invests in psychosocial prehabilitation [26,27,28].

Several limitations should be acknowledged. First, this was a single-center retrospective study, and SIPAT scoring was conducted by a specialized team within a particular institutional culture that is explicitly oriented toward rehabilitation rather than exclusion; generalizability to more restrictive programs is uncertain [13,14,15,25,26,27,28]. Second, we did not have standardized, coded reasons for non-listing and could not reliably disentangle “pure” psychosocial disqualifications from medical or logistical factors. In addition, standardized indicators of disease severity or transplant urgency were not consistently available for all candidates and, therefore, could not be included in all adjusted analyses. Because clinical urgency and disease severity may influence both psychosocial functioning and listing decisions, residual confounding cannot be excluded. Third, we examined only a single baseline SIPAT; longitudinal changes in psychosocial risk and the impact of specific interventions were not captured and may be much more informative than a single assessment in a program that actively modifies risk over time.

Importantly, we also considered whether baseline SIPAT scores might be associated with post-transplant mortality among transplanted recipients. However, only nine deaths occurred in this cohort during the observation period, providing too few events for any meaningful or stable predictive modeling. Any regression estimates in this context would be highly unreliable and at high risk of overfitting. For this reason, we deliberately chose not to present these exploratory checks in detail and do not interpret them as evidence for or against the prognostic value of SIPAT with respect to survival. Larger, prospective studies with sufficient numbers of events are needed to address the relationship between psychosocial risk and mortality in a robust way [19,20,21,22,23,24].

We also acknowledge that the present study focused primarily on transplant pathway outcomes rather than on the long-term effectiveness of psychosocial prehabilitation after transplantation. Standardized data regarding rehospitalizations, behavioral health utilization, adherence trajectories, and relapse rates were not consistently available for the entire cohort. Future prospective studies should examine whether targeted psychosocial prehabilitation reduces long-term psychosocial and medical risk among initially higher-risk candidates.

Finally, although our models included key psychosocial and demographic covariates, pseudo-*R*^2^ values were small and classification performance modest, indicating that many unmeasured clinical and contextual variables contribute to listing decisions. Our results should therefore be interpreted as describing how SIPAT is used within a specific, support-oriented program rather than as establishing universal thresholds or rules for candidate selection.

### 4.5. Clinical and Research Implications

Despite these limitations, our findings have several important implications. They provide empirical support for using SIPAT as a structured needs-assessment and psychosocial prehabilitation tool rather than as a rigid exclusion criterion [13,19,20,21,22,23,24,26,27,28]. Programs that adopt a similar philosophy may reasonably expect that higher SIPAT scores will flag patients who require more intensive psychosocial work, without necessarily condemning them to permanent non-listing. At the same time, our data underscore the ethical and clinical value of investing in psychosocial rehabilitation: high-risk patients in this cohort did not simply disappear from consideration; many progressed to listing despite elevated initial risk scores, particularly when substance-use and social problems were actively addressed.

For future research, our results suggest that work on SIPAT should move beyond a simple reliance on fixed cut-offs  and toward dynamic, multistage models. Combining baseline SIPAT with repeated follow-up assessments, adherence trajectories, clearly documented psychosocial interventions, and detailed clinical endpoints (including survival) may yield richer predictive frameworks for both listing and post-transplant outcomes. Finally, our findings support ongoing calls within transplantation to view psychosocial risk as modifiable and relational, rather than as a fixed property of the patient [13,14,15,25,26,27,28]. Tools like SIPAT can be powerful in this context, but only if they are used to open doors to support, not to close doors to care.

Our findings should therefore not be interpreted as evidence that psychosocial risk is unimportant or that SIPAT lacks clinical utility. Rather, they suggest that the meaning and predictive role of SIPAT depend substantially on how transplant programs operationalize psychosocial intervention, support allocation, and prehabilitation in routine practice.

## Figures and Tables

**Table 1:** Sociodemographic and clinical characteristics (*N* = 491).

Characteristic	Category/Value	*n*	%
**Age (years)**	Mean ± SD (range)	57.20 ± 10.73 (19–78)	-
**Sex**	Female	199	40.5
	Male	292	59.5
**Underlying diagnosis**	COPD/emphysema (incl. A1AT, asthma-COPD)	165	33.6
	Idiopathic/nonspecific ILD (incl. IPF)	121	24.6
	Pulmonary arterial/chronic pulmonary hypertension	40	8.1
	ILD in connective tissue disease (CTD-ILD)	36	7.3
	Hypersensitivity pneumonitis (HP)	30	6.1
	Post-COVID chronic lung disease (ILD/mixed)	21	4.3
	Sarcoidosis (± other)	17	3.5
	Other/mixed (cardiac, oncologic, etc.)	17	3.5
	Bronchiectasis (non-CF)	10	2.0
	Pneumoconiosis	8	1.6
	LAM	5	1.0
	Asthma (without clear COPD/ILD)	5	1.0
	Cystic fibrosis/CF/bronchiectasis	3	0.6
	Other rare ILD (e.g., LIP)	1	0.2
	Missing/not specified	12	2.4

Abbreviations: SD, standard deviation; COPD, chronic obstructive pulmonary disease; ILD, interstitial lung disease; IPF, idiopathic pulmonary fibrosis; CTD-ILD, connective tissue disease-related interstitial lung disease; HP, hypersensitivity pneumonitis; A1AT, alpha 1 antitrypsin deficiency; CF, cystic fibrosis; LAM, lymphangioleiomyomatosis; LIP, lymphocytic interstitial pneumonia; COVID, coronavirus disease 2019.

**Table 2:** SIPAT scores, psychosocial risk indicators, and listing status.

Variable	Total Sample (*N* = 491)	Non-Listed (*n* = 340)	Listed (*n* = 151)	*p*-Value
**Age, years, M ± SD**	57.20 ± 10.73	58.35 ± 10.43	54.60 ± 10.98	<0.001
**SIPAT total score, M ± SD**	12.38 ± 6.84	12.39 ± 6.95	12.37 ± 6.60	0.763
Readiness and illness management, M ± SD	4.25 ± 2.55	4.26 ± 2.61	4.23 ± 2.43	0.808
Social support, M ± SD	2.29 ± 2.27	2.30 ± 2.27	2.25 ± 2.26	0.886
Psychological stability/psychopathology, M ± SD	3.80 ± 3.16	3.72 ± 3.09	3.98 ± 3.31	0.428
Lifestyle/substance use, M ± SD	2.05 ± 2.60	2.11 ± 2.71	1.91 ± 2.32	0.751
**SIPAT global category, *n* (%)**				
Excellent candidate	90 (18.3)	59 (17.4)	31 (20.5)	—
Good candidate	349 (71.1)	244 (71.8)	105 (69.5)	—
Minimally acceptable candidate	48 (9.8)	33 (9.7)	15 (9.9)	—
Poor candidate	4 (0.8)	4 (1.2)	0 (0.0)	—
High-risk candidate	0 (0.0)	0 (0.0)	0 (0.0)	—
**Substance-related risk indicators, *n* (%)**				
Elevated alcohol-related risk	22 (4.5)	18 (5.3)	4 (2.6)	0.191
Elevated nicotine-related risk	56 (11.4)	36 (10.6)	20 (13.2)	0.393
Elevated illicit-substance-related risk	11 (2.2)	10 (2.9)	1 (0.7)	0.115

Table note: Continuous variables were compared using Mann–Whitney U tests. Categorical variables were compared using chi-square tests or Fisher’s exact test where appropriate. M, mean; SD, standard deviation; SIPAT, Stanford Integrated Psychosocial Assessment for Transplantation.

**Table 3:** Receiver operating characteristic analyses for SIPAT total and domain scores.

Variable	AUC	95% CI
SIPAT total	0.51	0.45–0.56
Readiness and illness management	0.51	0.45–0.56
Social support	0.50	0.44–0.55
Psychological stability/psychopathology	0.52	0.47–0.58
Lifestyle/substance use	0.51	0.46–0.56

**Table 4:** Multivariable logistic regression models predicting transplant listing status.

Predictor	Model 1 OR (95% CI), *p*	Model 2 OR (95% CI), *p*	Model 3 OR (95% CI), *p*
Age	0.968 (0.951–0.986), *p* < 0.001	0.968 (0.951–0.986), *p* < 0.001	0.966 (0.948–0.983), *p* < 0.001
Sex	1.139 (0.766–1.703), *p* = 0.522	1.228 (0.815–1.861), *p* = 0.329	1.199 (0.802–1.802), *p* = 0.380
SIPAT total	0.995 (0.967–1.024), *p* = 0.742	—	—
Readiness and illness management	—	0.988 (0.907–1.073), *p* = 0.768	—
Social support	—	0.990 (0.904–1.083), *p* = 0.828	—
Psychological stability	—	1.032 (0.965–1.105), *p* = 0.356	—
Lifestyle/substance use	—	0.947 (0.868–1.027), *p* = 0.203	—
Alcohol-related risk	—	—	0.406 (0.111–1.173), *p* = 0.124
Nicotine-related risk	—	—	1.400 (0.738–2.602), *p* = 0.293
Illicit substance-related risk	—	—	0.177 (0.009–1.054), *p* = 0.117
**Model statistic**			
Classification accuracy	68.7%	69.9%	69.3%
McFadden pseudo-R^2^	0.021	0.025	0.034
Nagelkerke pseudo-R^2^	0.037	0.043	0.058

**Table note:** *Model 1 included age, sex, and SIPAT total score.* Model 2 included age, sex, and four SIPAT domain scores. Model 3 included age, sex, and binary substance-related risk indicators. OR, odds ratio; CI, confidence interval.

## Data Availability

The data presented in this study are available on reasonable request from the corresponding author. The data are not publicly available due to privacy and ethical restrictions related to the use of clinical patient data.

## Abbreviations

The following abbreviations are used in this manuscript:

A1AT	alpha-1 antitrypsin deficiency
AUC	area under the curve (receiver operating characteristic)
CF	cystic fibrosis
CI	confidence interval
COPD	chronic obstructive pulmonary disease
COVID-19	coronavirus disease 2019
CTD-ILD	connective tissue disease-related interstitial lung disease
HP	hypersensitivity pneumonitis
ILD	interstitial lung disease
IQR	interquartile range
LAM	lymphangioleiomyomatosis
LIP	lymphocytic interstitial pneumonia
M	mean
OR	odds ratio
ROC	receiver operating characteristic
SD	standard deviation
SIPAT	Stanford Integrated Psychosocial Assessment for Transplant
